# Supplementary figures and images for: Two novel loci underlie natural differences in Caenorhabditis elegans abamectin responses
Source: PLoS Pathog. 2021 Mar 15;17(3):e1009297. doi: 10.1371/journal.ppat.1009297 (PMC7993787; doi:10.1371/journal.ppat.1009297)

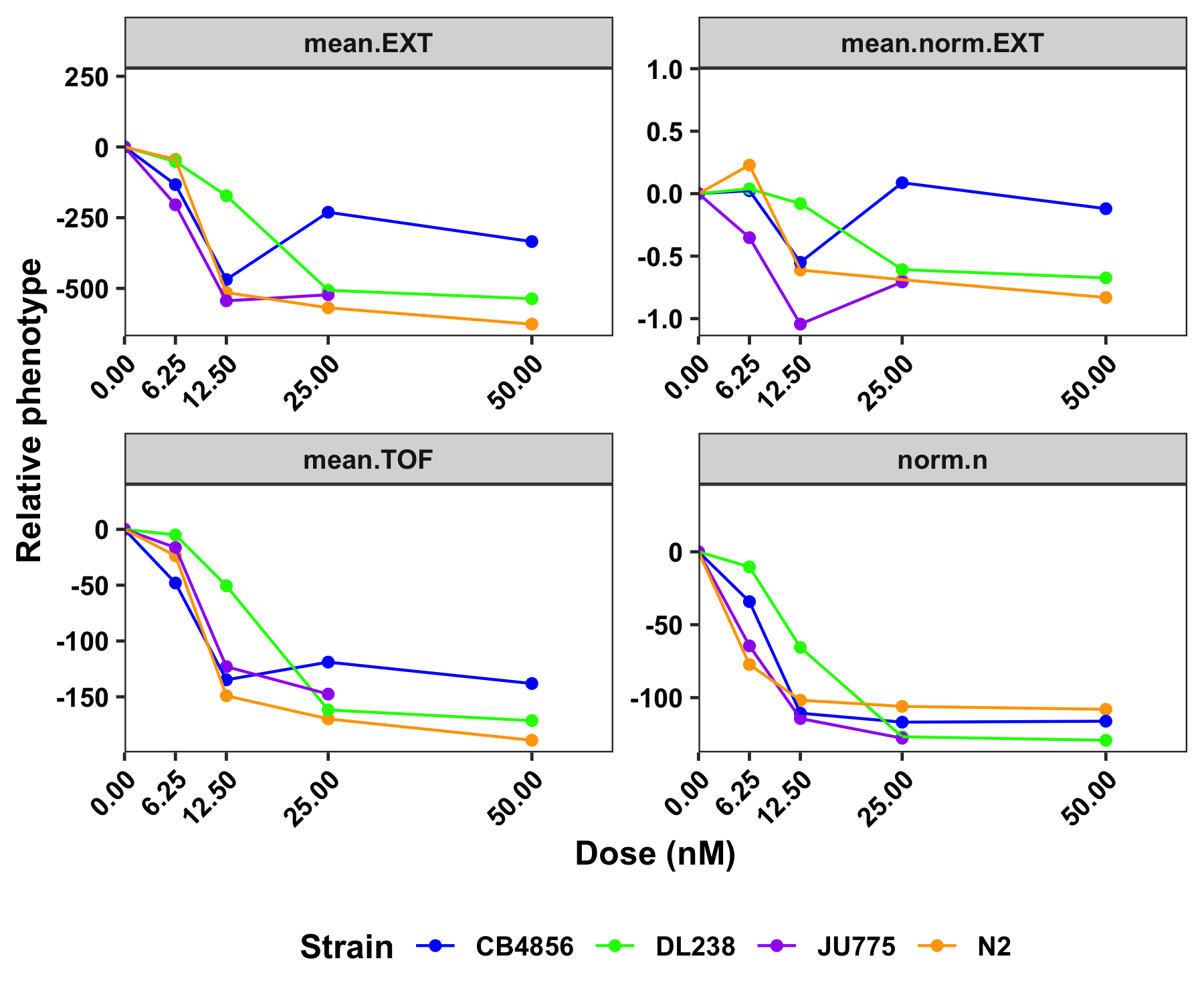

Supplement: S1 Fig — Results from the abamectin dose response HTA for brood size (norm.n), animal length (mean.TOF), animal optical density (mean.EXT), and normalized optical density (mean.norm.EXT) are shown. For each trait, drug concentration (nM) (x-axis) is plotted against phenotype subtracted from control (y-axis) and colored by strain (CB4856: blue, DL238: green, JU775: purple, N2: orange). A concentration of 7.5 nM was chosen for future experiments. (PNG) [file ppat.1009297.s001.png]

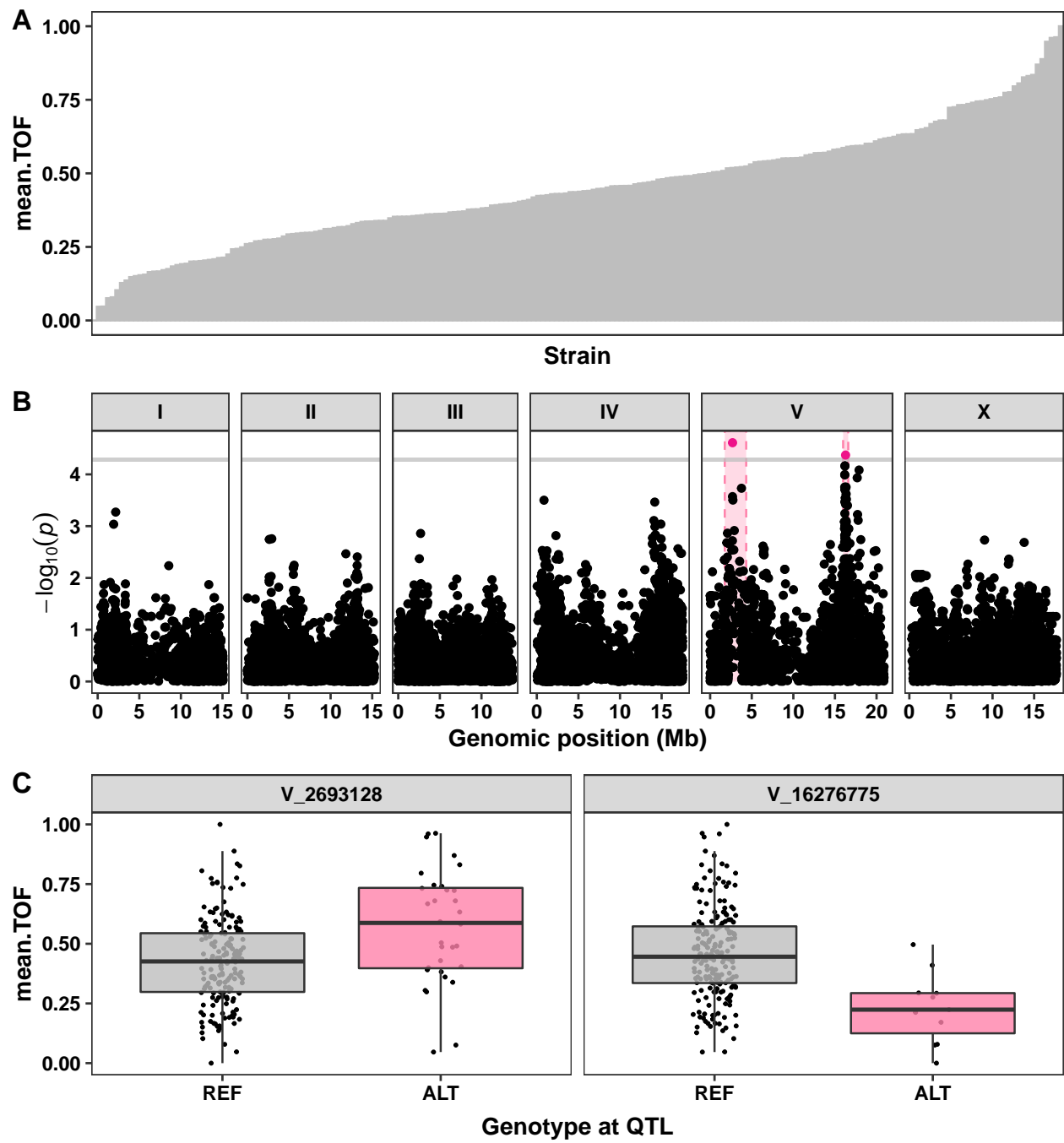

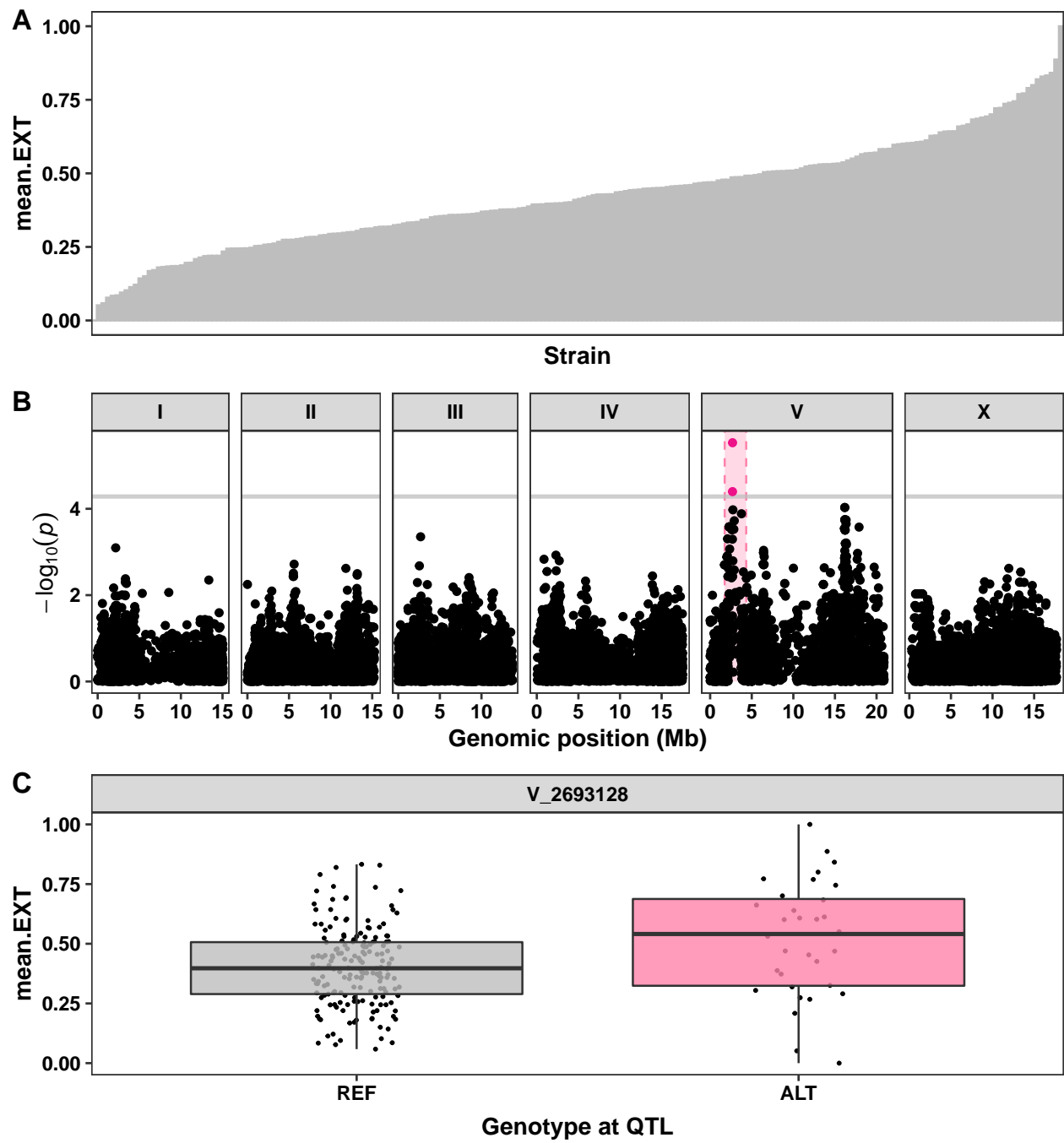

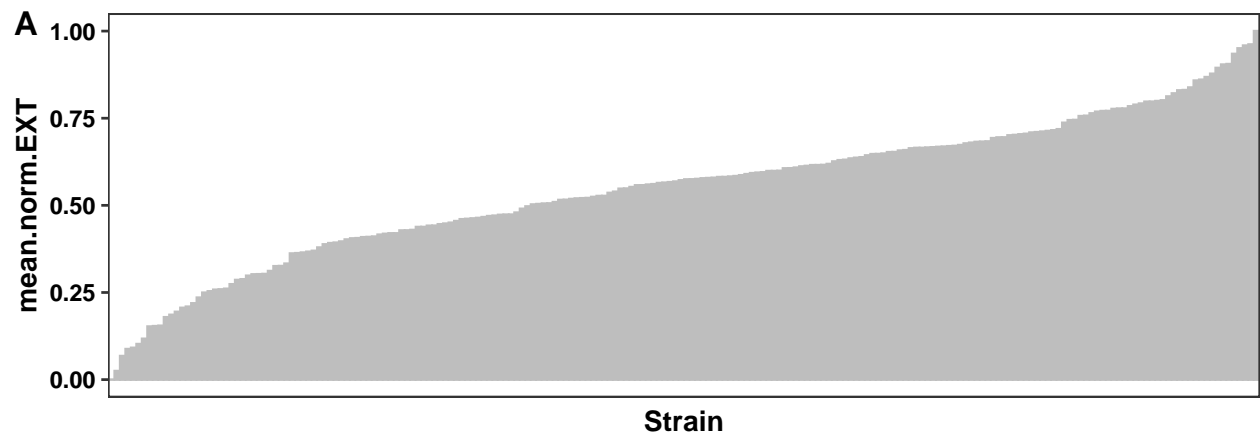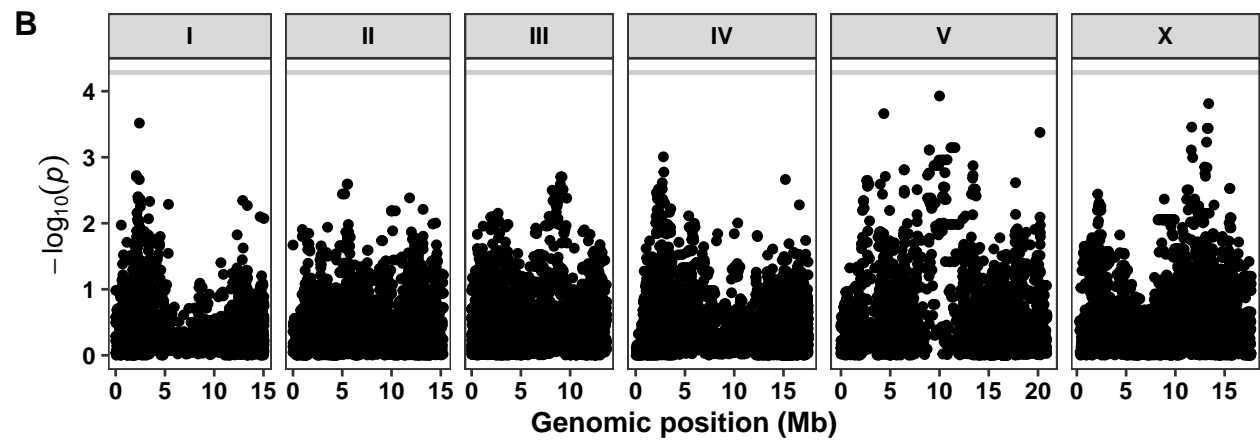

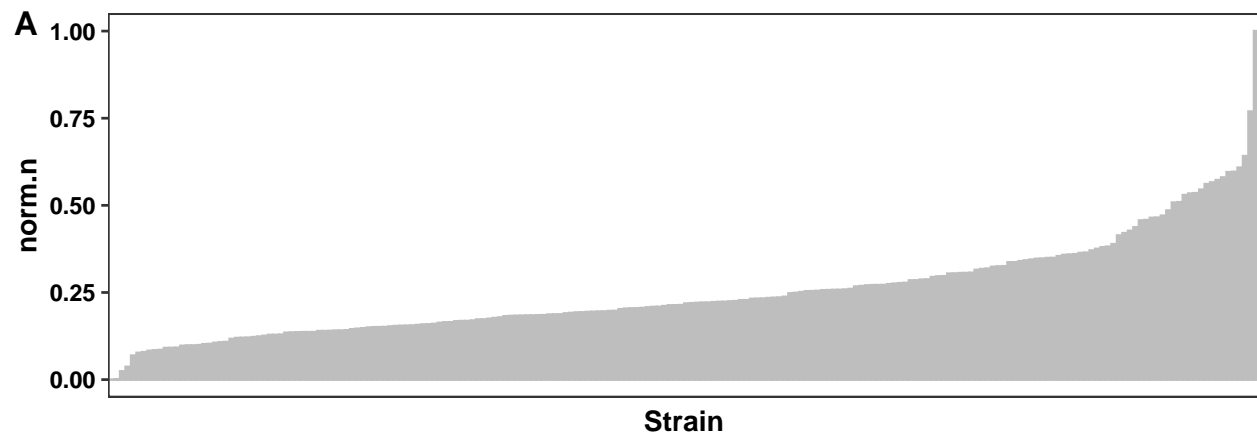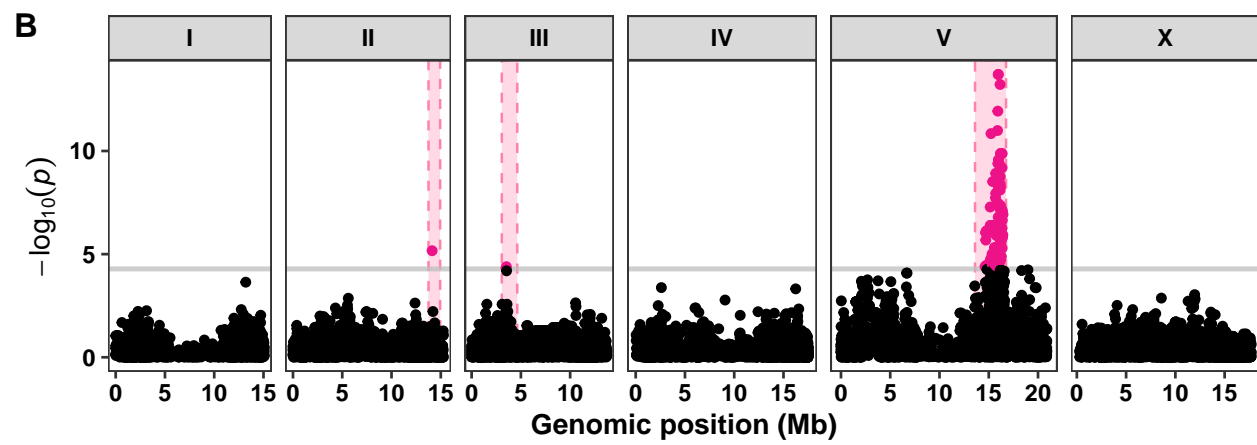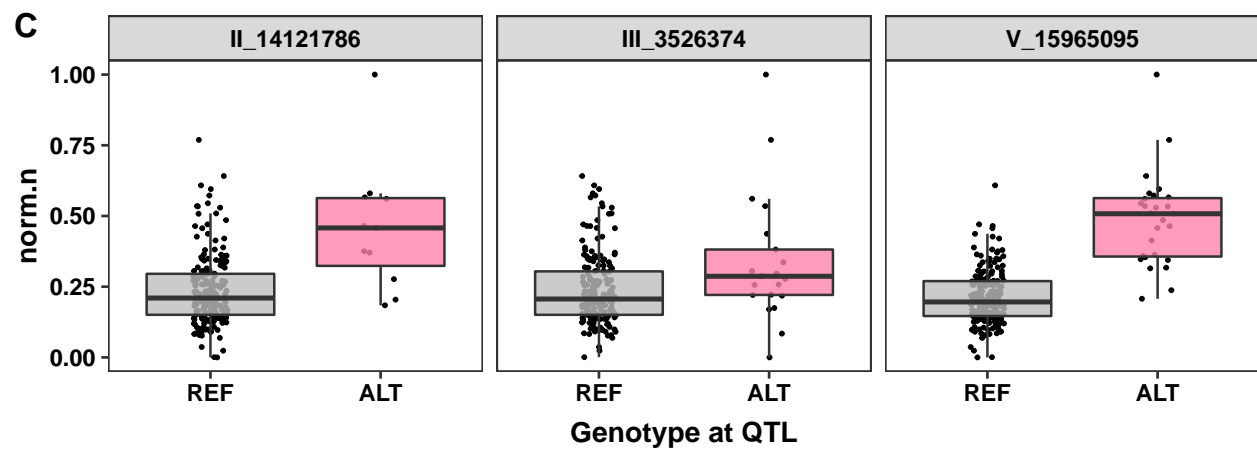

Supplement: S2 Fig — A) Normalized residual phenotype (y-axis) of 210 wild isolates (x-axis) in response to abamectin. B) Association mapping results are shown. Genomic position (x-axis) is plotted against the -log10(p) value (y-axis) for each SNV. SNVs are colored pink if they pass the genome-wide eigen-decomposition significance threshold designated by the grey line. The genomic regions of interest that pass the significance threshold are highlighted by pink rectangles. C) For each QTL, the normalized residual phenotype (y-axis) of strains split by genotype at the peak marker (x-axis) are plotted as Tukey box plots. Each point corresponds to a wild isolate strain. Strains with the N2 reference allele are colored grey, and strains with an alternative allele are colored pink. (PDF) [file ppat.1009297.s002.pdf]

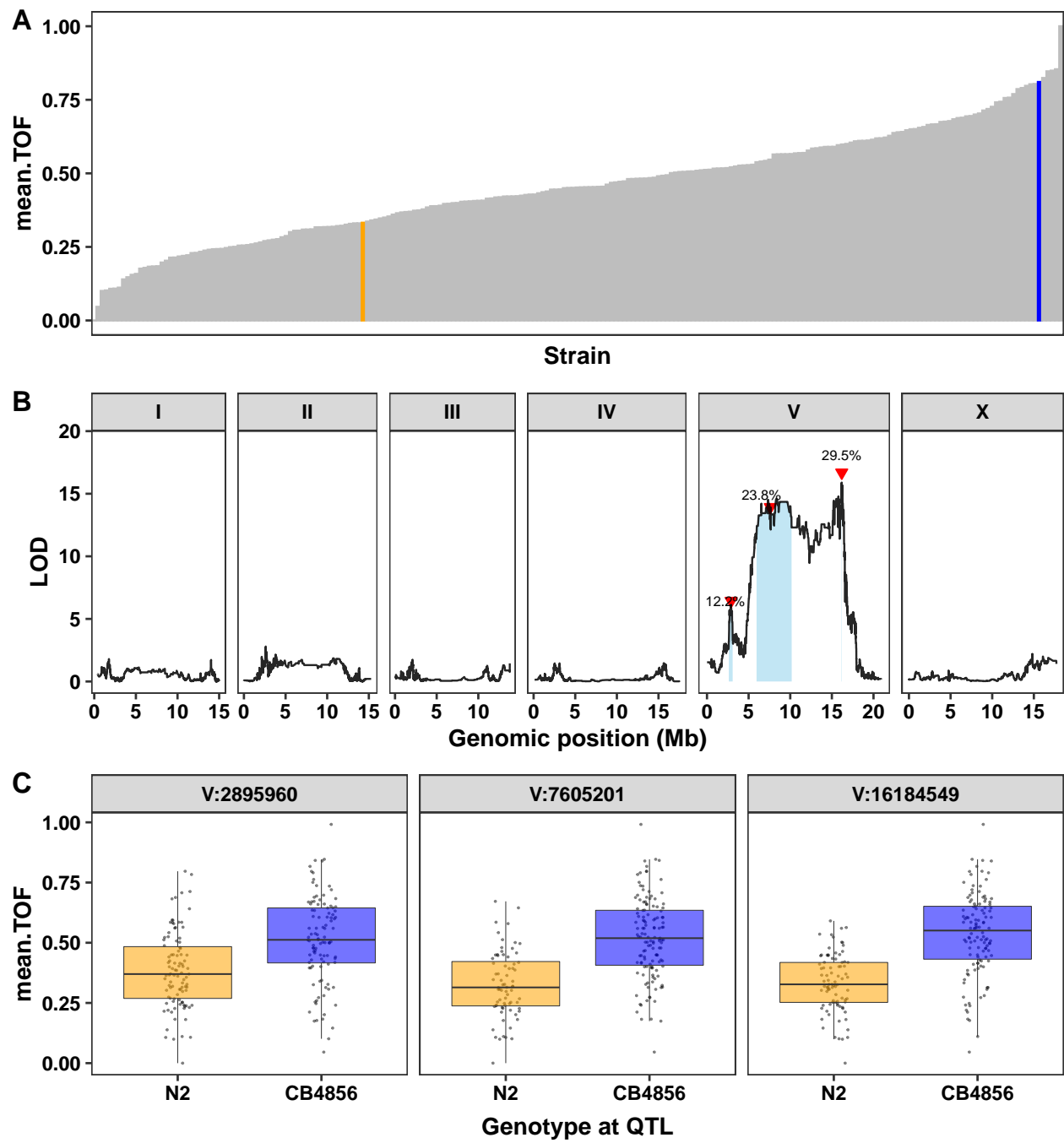

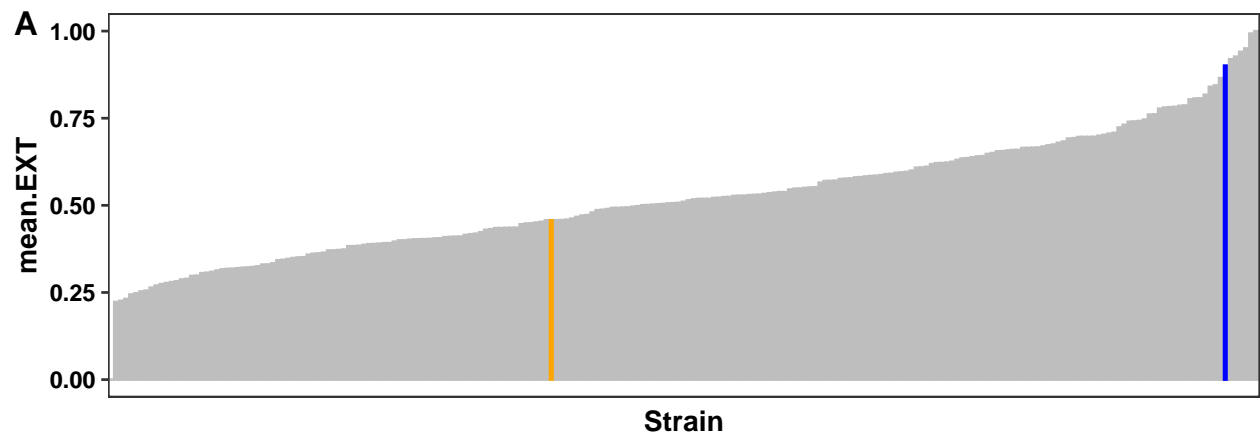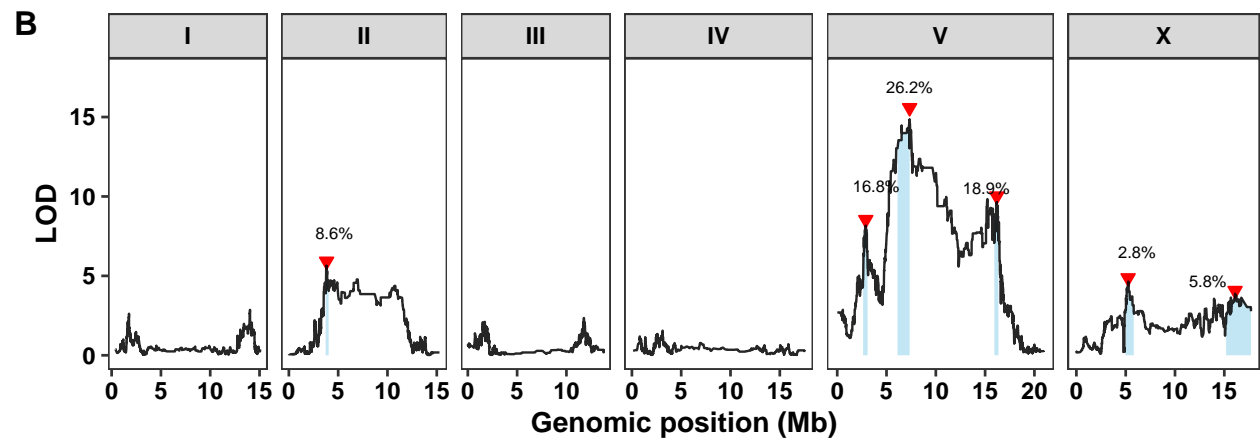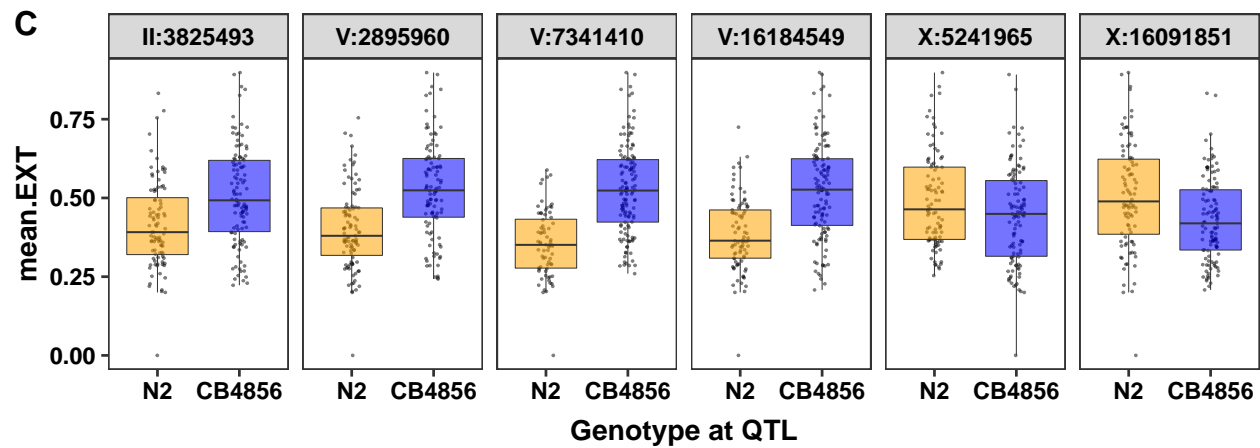

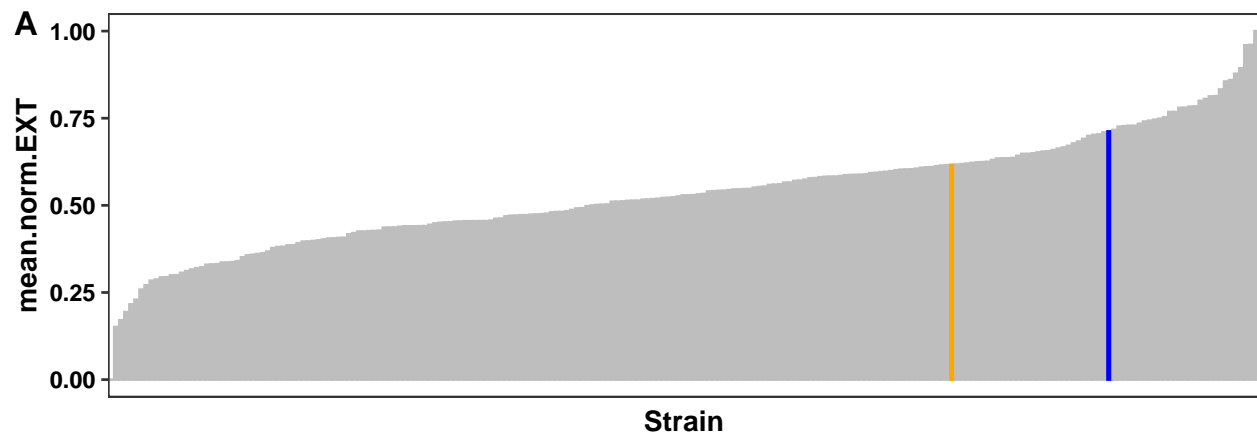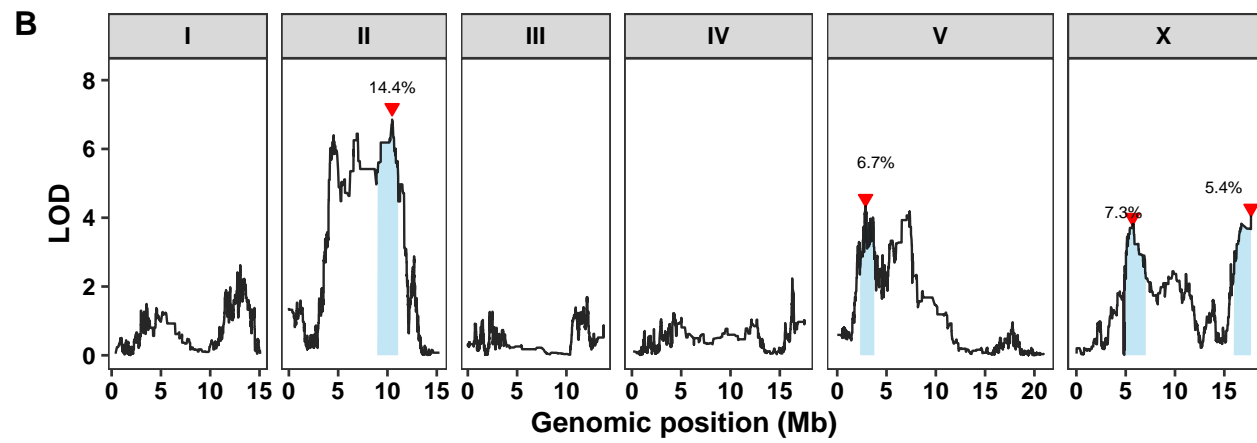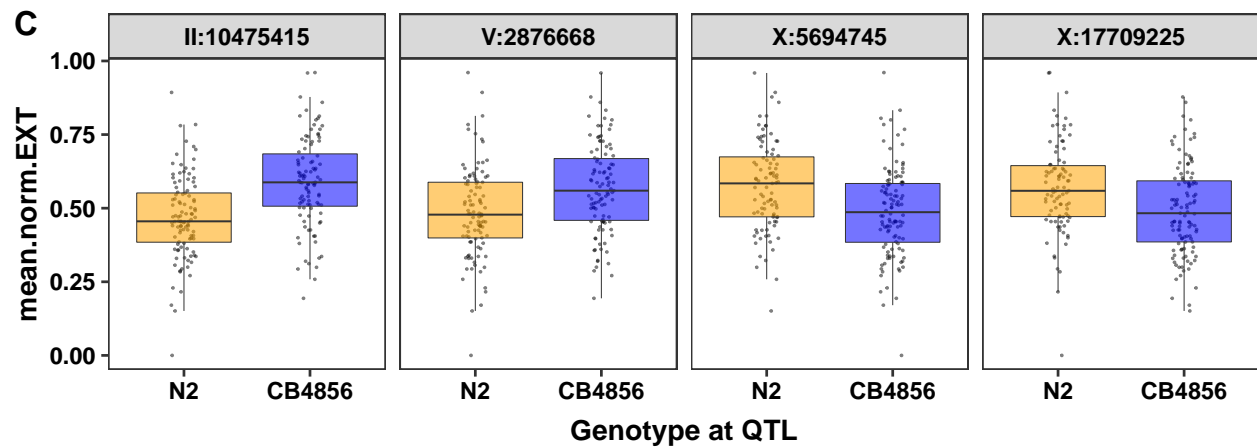

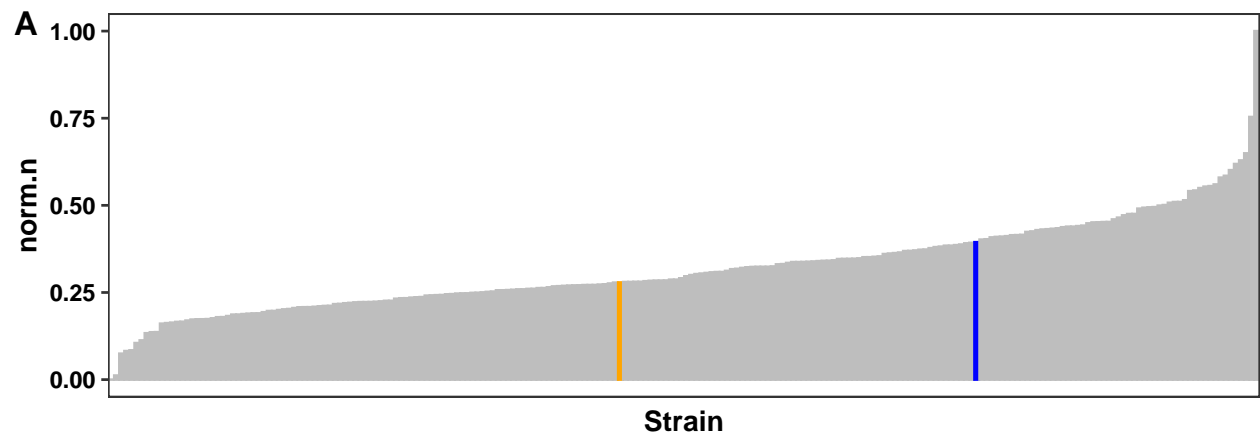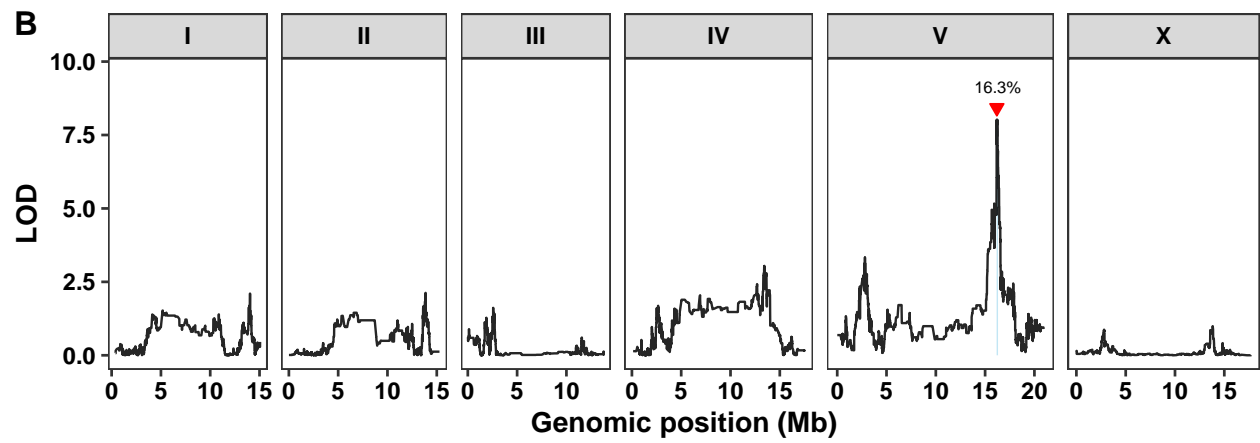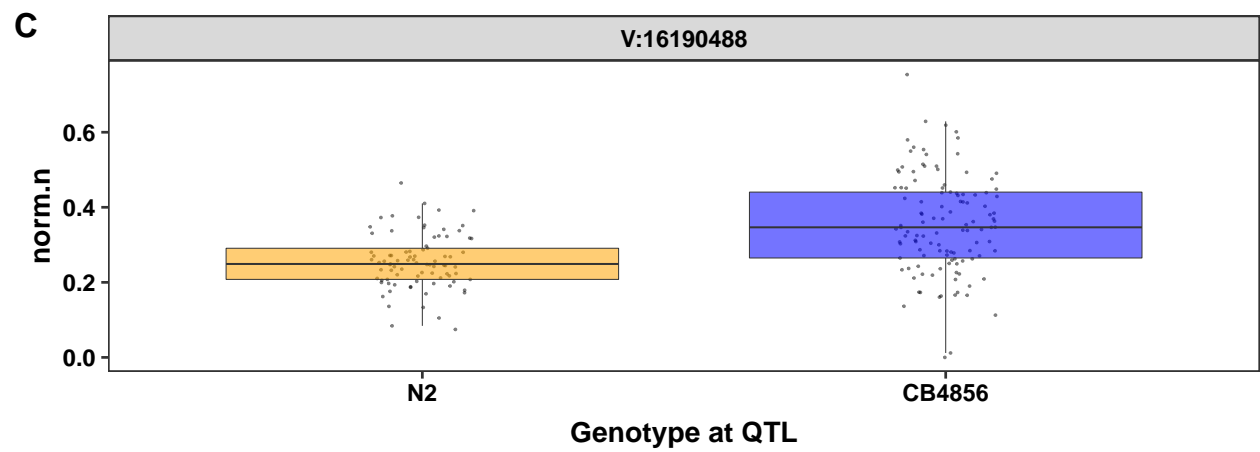

Supplement: S3 Fig — A) Normalized residual phenotype (y-axis) of 225 RIAILs (x-axis) in response to abamectin. The parental strains are colored: N2, orange; CB4856, blue. B) Linkage mapping results are shown. Genomic position in Mb (x-axis) is plotted against the logarithm of the odds (LOD) score (y-axis) for 13,003 genomic markers. Each significant QTL is indicated by a red triangle at the peak marker, and a blue rectangle shows the 95% confidence interval around the peak marker. The percentage of the total variance in the RIAIL population that can be explained by each QTL is shown above the QTL. C) For each QTL, the normalized residual phenotype (y-axis) of RIAILs split by genotype at the marker with the maximum LOD score (x-axis) are plotted as Tukey box plots. Each point corresponds to a unique recombinant strain. Strains with the N2 allele are colored orange, and strains with the CB4856 allele are colored blue. (PDF) [file ppat.1009297.s003.pdf]

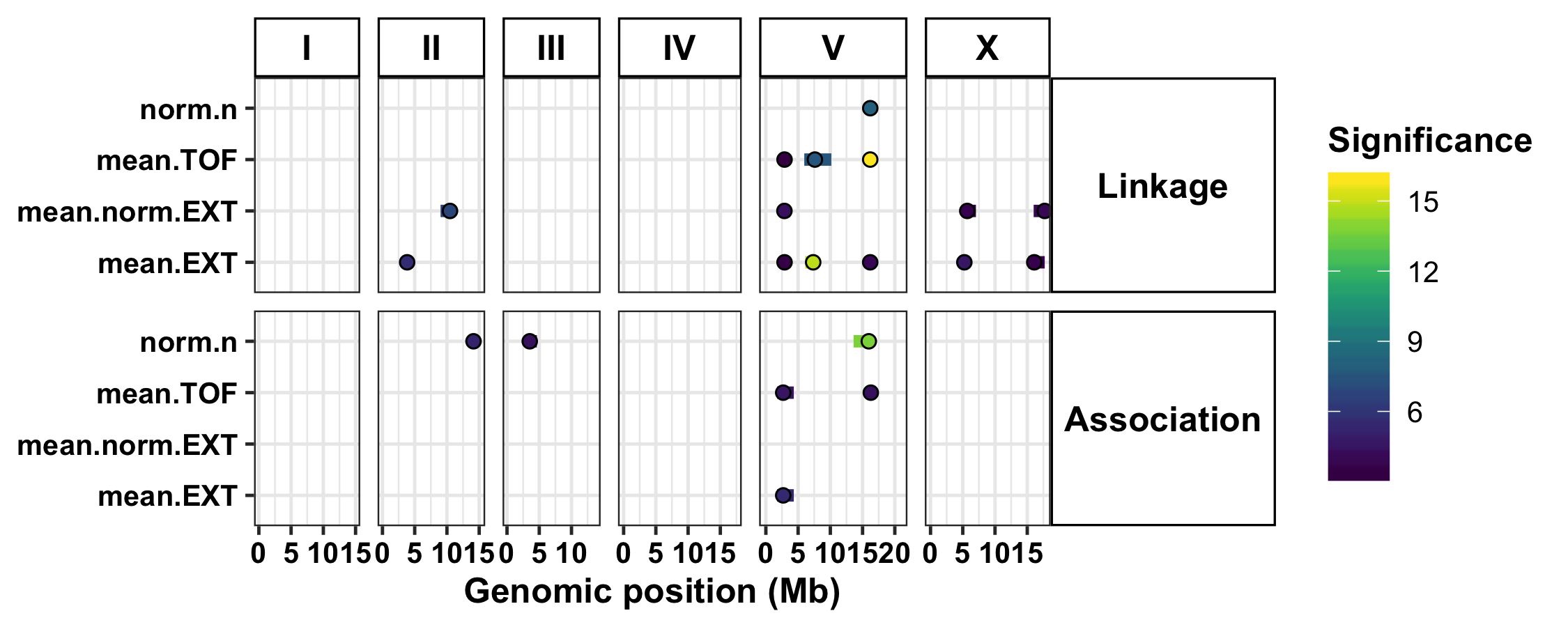

Supplement: S4 Fig — Genomic positions (x-axis) of all QTL identified from linkage mapping (top) and association mapping (bottom) are shown for each drug-trait (y-axis). Each QTL is plotted as a point at the genomic location of the peak marker and a line that represents the confidence interval. QTL are colored by the significance of the LOD score (linkage) or -log10(p) value (association), increasing from purple to green to yellow. (PNG) [file ppat.1009297.s004.png]

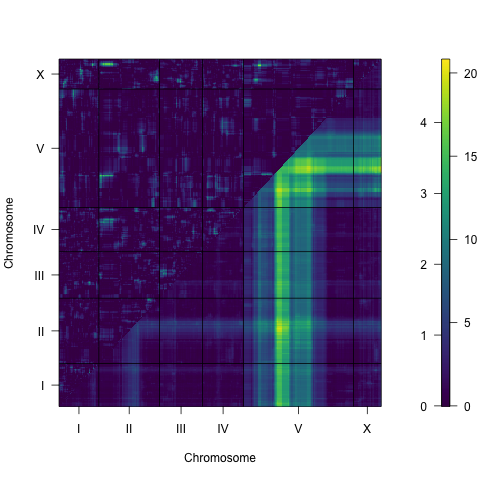

Supplement: S5 Fig — Log of the odds (LOD) scores are shown for each pairwise combination of loci, split by chromosome. The upper-left triangle contains the epistasis LOD scores (interaction effects), and the lower-right triangle contains the LOD scores for the full model (both interaction and additive effects). LOD scores are colored by significance, increasing from purple to green to yellow. The LOD scores for the epistasis model are shown on the left of the color scale, and the LOD scores for the full model are shown on the right. (PNG) [file ppat.1009297.s005.png]

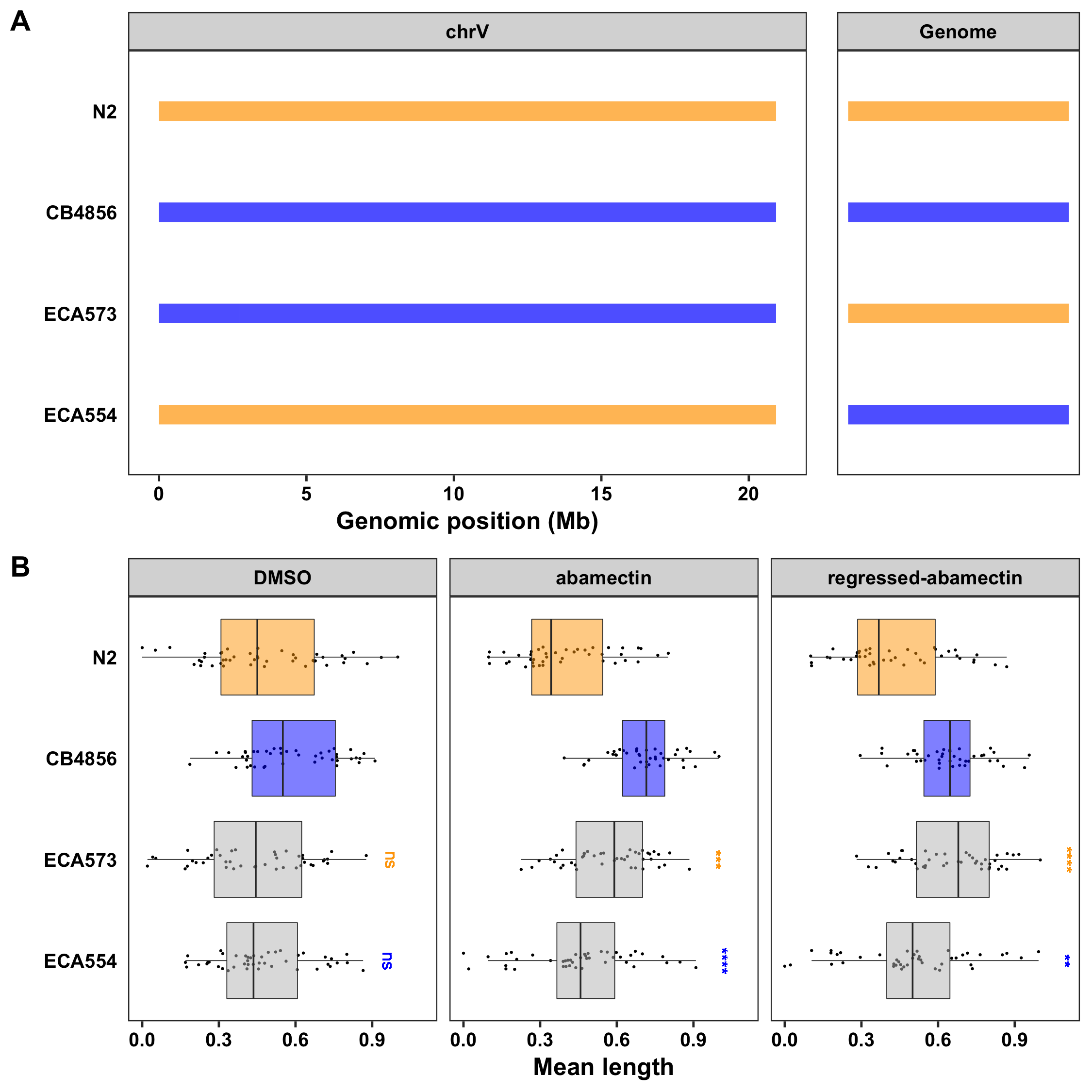

Supplement: S6 Fig — A) Strain genotypes are shown as colored rectangles (N2: orange, CB4856: blue) in detail for chromosome V (left) and in general for the rest of the chromosomes (right). B) Animal lengths (mean.TOF, x-axis) are plotted as Tukey box plots against strain (y-axis) for DMSO control (left), abamectin (center), and abamectin regressed by DMSO (right). Statistical significance of each NIL as compared to its parental strain (ECA573 to N2 and ECA554 to CB4856) calculated by Tukey’s HSD is shown above each strain (ns = non-significant (p-value > 0.05); *, **, ***, and *** = significant (p-value < 0.05, 0.01, 0.001, or 0.0001, respectively). (PNG) [file ppat.1009297.s006.png]

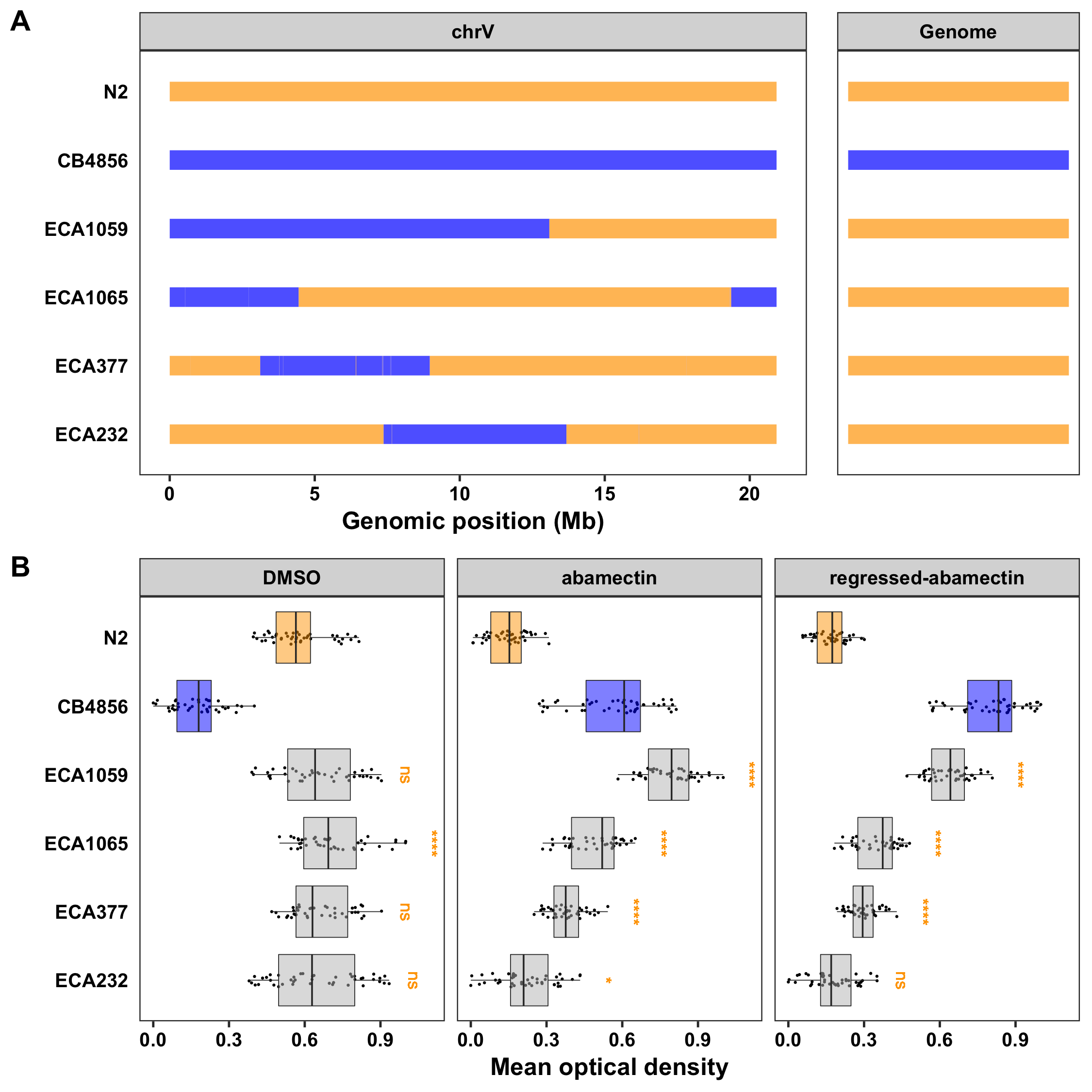

Supplement: S7 Fig — A) Strain genotypes are shown as colored rectangles (N2: orange, CB4856: blue) in detail for chromosome V (left) and in general for the rest of the chromosomes (right). B) Animal optical densities (mean.EXT, x-axis) are plotted as Tukey box plots against strain (y-axis) for DMSO control (left), abamectin (center), and abamectin regressed by DMSO (right). Statistical significance of each NIL as compared to its parental strain (ECA573 to N2 and ECA554 to CB4856) calculated by Tukey’s HSD is shown above each strain (ns = non-significant (p-value > 0.05); *, **, ***, and *** = significant (p-value < 0.05, 0.01, 0.001, or 0.0001, respectively). (PNG) [file ppat.1009297.s007.png]

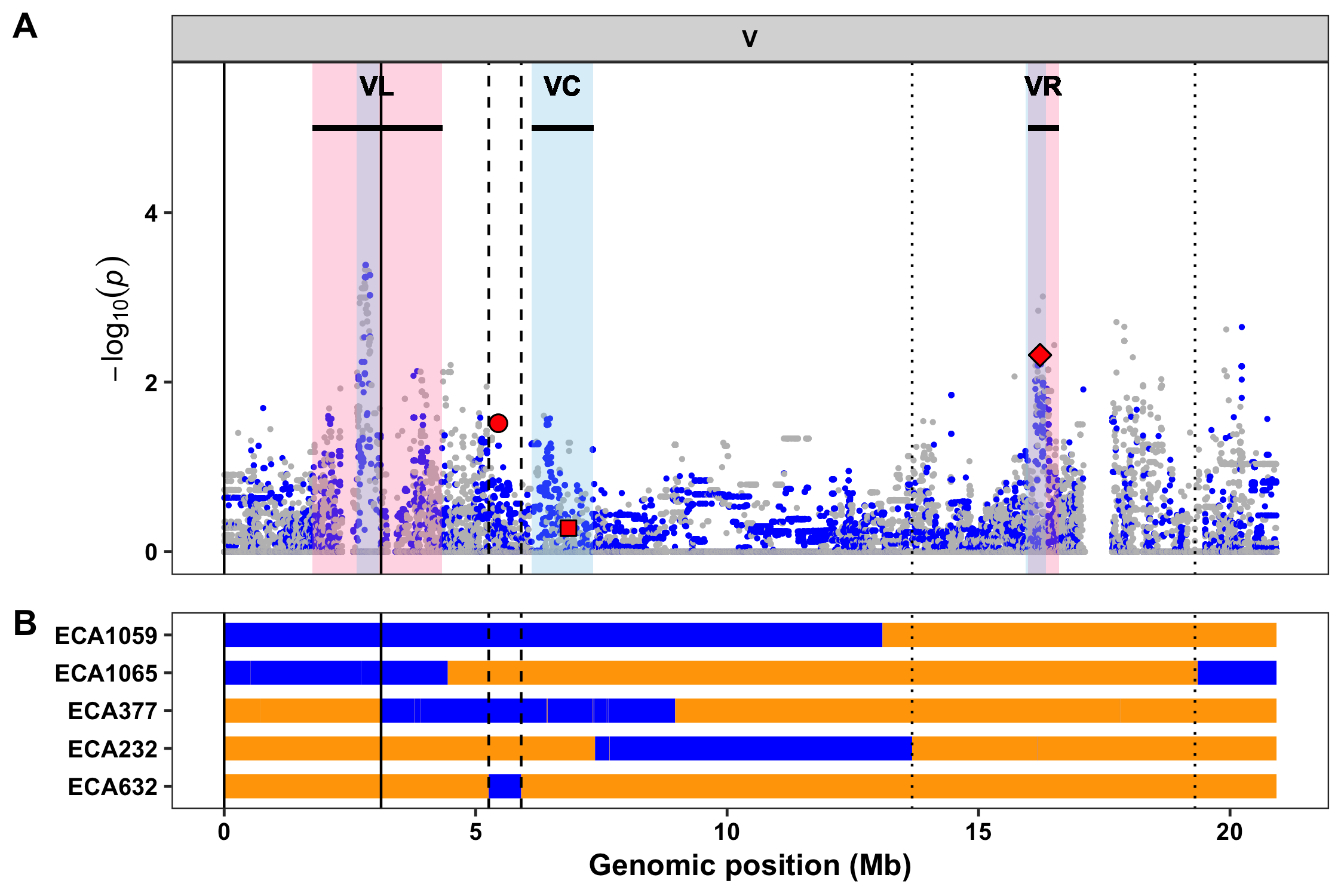

Supplement: S8 Fig — A) Fine mapping of all common variants on chromosome V is shown. Genomic position (x-axis) is plotted against the -log10(p) values (y-axis) for each variant and colored by the genotype of the variant in the CB4856 strain (grey = N2 reference allele, blue = variation from the N2 reference allele). Genomic regions identified from linkage mapping analysis are highlighted in blue and genomic regions identified from association mapping are highlighted in pink. The horizontal grey line represents the genome-wide eigen-decomposition significance threshold. The red points represent the positions of the most significant variants in the genes glc-1 (diamond), glc-3 (circle), and lgc-54 (square). The vertical lines represent the smallest NIL-defined genomic region for the VL (solid), VC (dashed), and VR (dotted) QTL. B) Strain genotypes are shown as colored rectangles (N2: orange, CB4856: blue) in detail for chromosome V. The vertical lines represent the smallest NIL-defined genomic region for the VL (solid), VC (dashed), and VR (dotted) QTL. (PNG) [file ppat.1009297.s008.png]

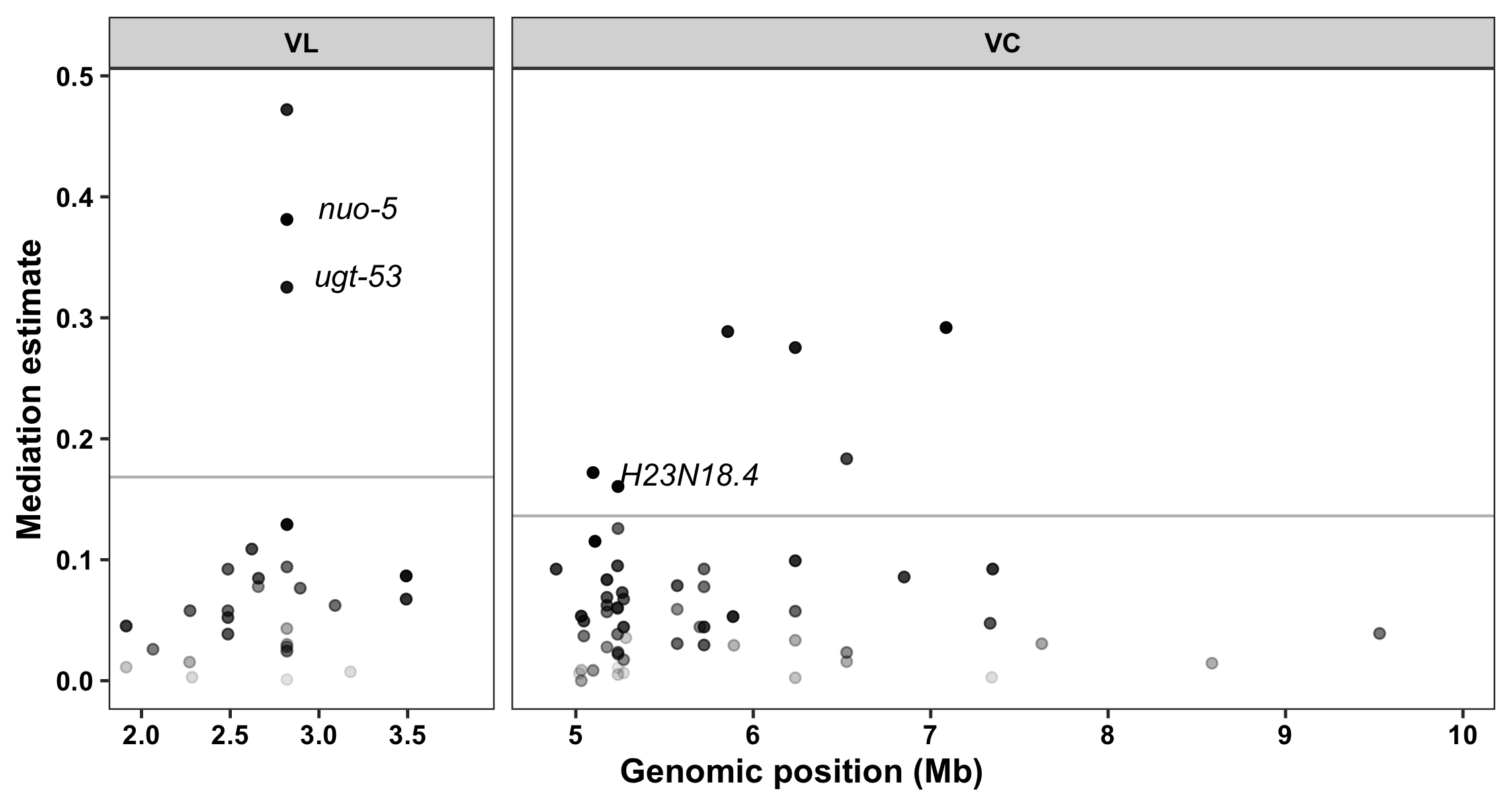

Supplement: S9 Fig — Mediation estimates calculated as the indirect effect that differences in expression of each gene plays in the overall phenotype (y-axis) are plotted against genomic position of the eQTL (x-axis) on chromosome V for all genes with a gene expression QTL in the narrowed VL and VC intervals. The 90th percentile of the distribution of mediation estimates is represented by the horizontal grey line. The confidence of the estimate increases (p-value decreases) as points become more solid. (PNG) [file ppat.1009297.s009.png]

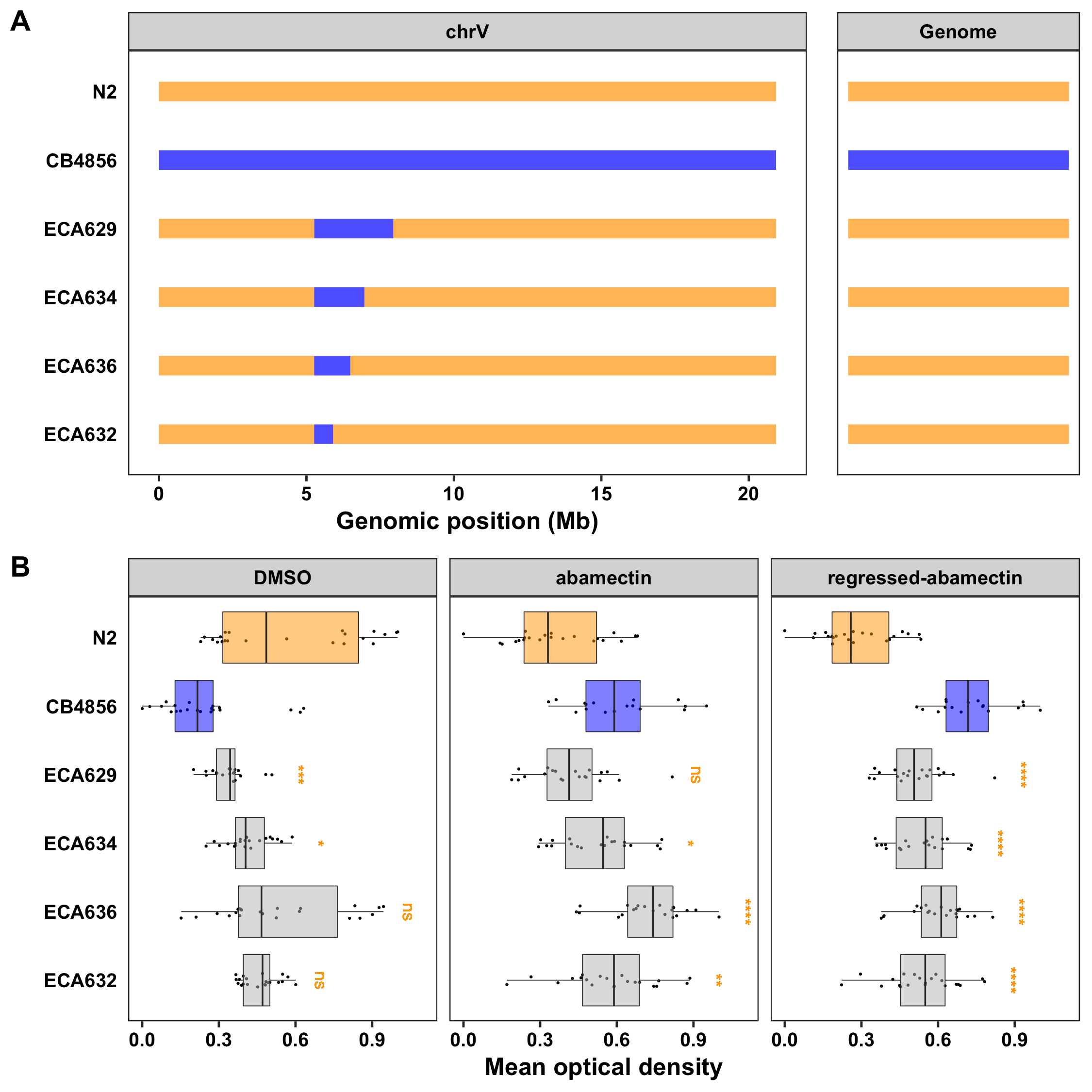

Supplement: S10 Fig — A) Strain genotypes are shown as colored rectangles (N2: orange, CB4856: blue) in detail for chromosome V (left) and in general for the rest of the chromosomes (right). B) Animal lengths (mean.TOF, x-axis) are plotted as Tukey box plots against strain (y-axis) for DMSO control (left), abamectin (center), and abamectin regressed by DMSO (right). Statistical significance of each NIL as compared to its parental strain (ECA573 to N2 and ECA554 to CB4856) calculated by Tukey’s HSD is shown above each strain (ns = non-significant (p-value > 0.05); *, **, ***, and *** = significant (p-value < 0.05, 0.01, 0.001, or 0.0001, respectively). (PNG) [file ppat.1009297.s010.png]

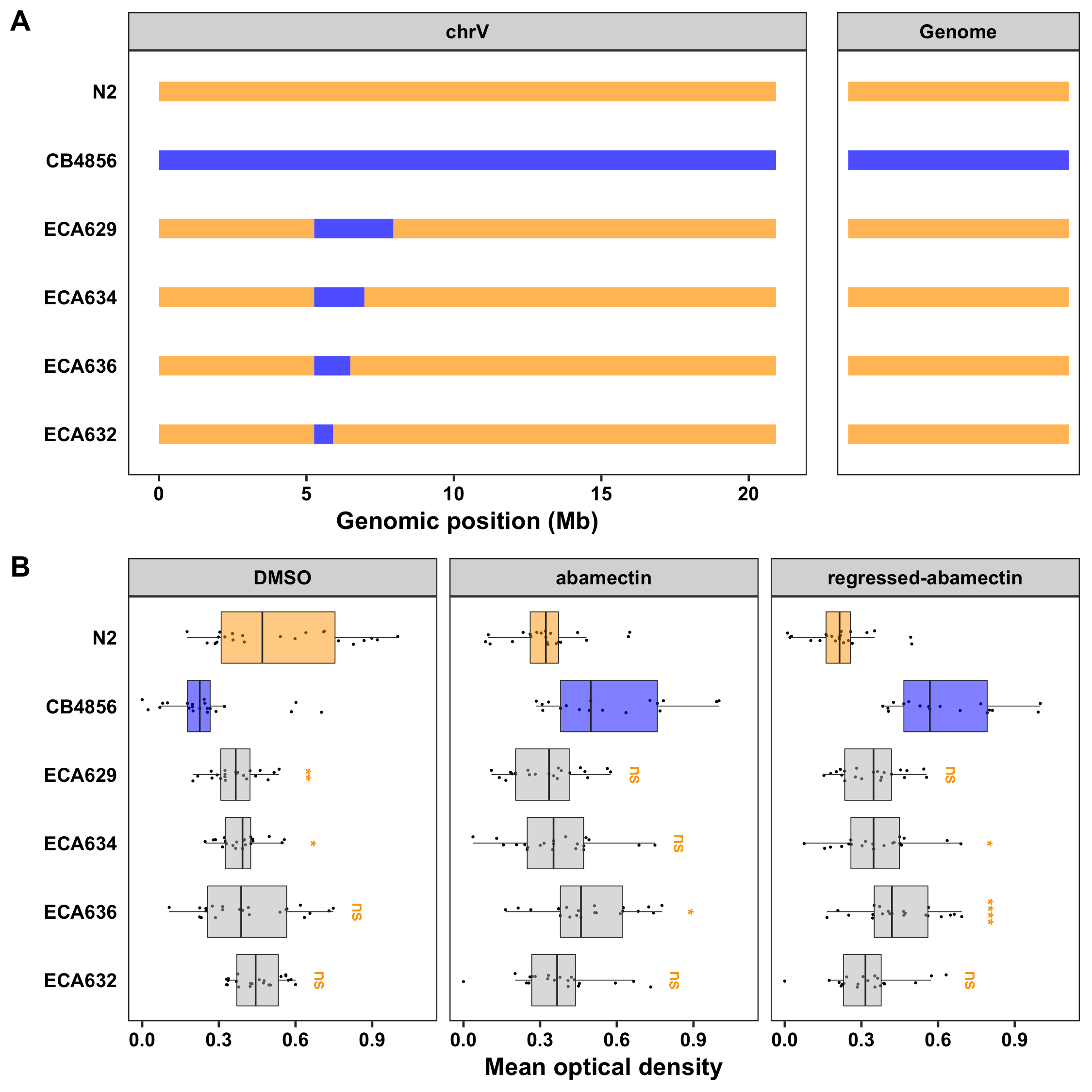

Supplement: S11 Fig — A) Strain genotypes are shown as colored rectangles (N2: orange, CB4856: blue) in detail for chromosome V (left) and in general for the rest of the chromosomes (right). The dashed vertical lines represent the previous NIL-defined QTL interval for VC. B) Animal optical densities (mean.EXT, x-axis) are plotted as Tukey box plots against strain (y-axis) for DMSO control (left), abamectin (center), and abamectin regressed by DMSO (right). Statistical significance of each NIL as compared to the N2 strain calculated by Tukey’s HSD is shown above each strain (ns = non-significant (p-value > 0.05); *, **, ***, and *** = significant (p-value < 0.05, 0.01, 0.001, or 0.0001, respectively). (PNG) [file ppat.1009297.s011.png]
