# Supplementary material for: Two novel loci underlie natural differences in Caenorhabditis elegans abamectin responses
Source: PLoS Pathog. 2021 Mar 15;17(3):e1009297. doi: 10.1371/journal.ppat.1009297 (PMC7993787; doi:10.1371/journal.ppat.1009297)
Supplement: S1 Text — Contains a list of all strains and primers used to genotype NILs. (PDF) [file ppat.1009297.s031.pdf]

Supplementary Material

Wild isolates used (GWAS):
AB1, BRC20067, BRC20263, CB4852, CB4854, CB4856, CB4932, CX11262, CX11264, CX11271, CX11276, CX11285, CX11292, CX11307, CX11314, CX11315, DL200, DL226, DL238, ECA189, ECA248, ECA250, ECA251, ECA348, ECA349, ECA36, ECA369, ECA372, ECA396, ED3005, ED3011, ED3017, ED3040, ED3046, ED3048, ED3049, ED3052, ED3073, ED3077, EG4347, EG4349, EG4725, EG4946, GXW1, JT11398, JU1088, JU1172, JU1212, JU1242, JU1246, JU1249, JU1395, JU1409, JU1440, JU1491, JU1543, JU1568, JU1580, JU1581, JU1652, JU1666, JU1792, JU1793, JU1808, JU1896, JU1934, JU2007, JU2016, JU2017, JU2106, JU2131, JU2141, JU2234, JU2250, JU2257, JU2464, JU2466, JU2513, JU2522, JU2526, JU2534, JU2565, JU2566, JU2570, JU2572, JU2575, JU2576, JU2578, JU258, JU2581, JU2586, JU2587, JU2592, JU2593, JU2600, JU2610, JU2619, JU2800, JU2811, JU2825, JU2829, JU2838, JU2841, JU2853, JU2862, JU2866, JU2878, JU2879, JU2906, JU2907, JU310, JU311, JU3125, JU3127, JU3128, JU3132, JU3134, JU3135, JU3137, JU3140, JU3144, JU323, JU346, JU360, JU367, JU393, JU394, JU397, JU406, JU440, JU561, JU642, JU751, JU774, JU775, JU778, JU782, JU792, JU830, KR314, LKC34, MY1, MY10, MY16, MY18, MY2147, MY2212, MY23, MY2530, MY2535, MY2573, MY2693, MY2713, MY2741, MY518, MY679, MY772, MY920, NIC1049, NIC1107, NIC166, NIC195, NIC199, NIC207, NIC231, NIC236, NIC242, NIC251, NIC252, NIC256, NIC258, NIC259, NIC260, NIC261, NIC262, NIC265, NIC266, NIC267, NIC268, NIC269, NIC271, NIC272, NIC274, NIC275, NIC276, NIC3, NIC501, NIC511, NIC514, NIC515, NIC522, NIC526, NIC527, PB303, PS2025, PX179, QG556, QG557, QX1212, QX1233, QX1791, QX1792, QX1793, QX1794, RC301, WN2001, WN2033, WN2050, XZ1513, XZ1514

RIAILs used:
<u>Set 2 (linkage mapping):</u>
QX297, QX364, QX299, QX311, QX358, QX511, QX314, QX565, QX304, QX448, QX557, QX401, QX243, QX417, QX503, QX453, QX561, QX403, QX437, QX352, QX420, QX418, QX426, QX506, QX529, QX538, QX553, QX245, QX491, QX279, QX315, QX521, QX456, QX563, QX465, QX372, QX449, QX334, QX378, QX497, QX435, QX371, QX540, QX321, QX336, QX270, QX432, QX332, QX254, QX406, QX528, QX275, QX484, QX597, QX438, QX520, QX459, QX476, QX337, QX452, QX554, QX559, QX348, QX341, QX373, QX295, QX429, QX322, QX393, QX514, QX386, QX326, QX436, QX355, QX380, QX328, QX325, QX574, QX360, QX473, QX467, QX517, QX253, QX274, QX466, QX477, QX240, QX349, QX394, QX242, QX244, QX474, QX293, QX523, QX490, QX560, QX376, QX271, QX549, QX594, QX531, QX513, QX261, QX539, QX441, QX573, QX339, QX479, QX362, QX570,

QX340, QX284, QX319, QX498, QX434, QX298, QX483, QX508, QX585, QX447, QX384, QX469, QX584, QX283, QX461, QX309, QX580, QX296, QX428, QX409, QX365, QX316, QX343, QX369, QX347, QX291, QX367, QX377, QX500, QX405, QX470, QX323, QX286, QX407, QX495, QX338, QX312, QX331, QX472, QX556, QX392, QX412, QX280, QX310, QX396, QX400, QX366, QX527, QX294, QX368, QX356, QX387, QX263, QX329, QX324, QX478, QX346, QX596, QX381, QX457, QX281, QX300, QX264, QX391, QX379, QX583, QX501, QX353, QX471, QX424, QX389, QX464, QX410, QX302, QX303, QX320, QX398, QX395, QX443, QX545, QX587, QX276, QX493, QX359, QX399, QX305, QX363, QX445, QX524, QX252, QX446, QX515, QX431, QX440, QX423, QX455, QX551, QX289, QX458, QX345, QX555, QX285, QX241, QX357, QX489, QX533, QX330, QX512, QX542, QX460, QX268, QX564, QX572, QX287, QX258

Set 1 (mediation analysis):

QX89, QX218, QX137, QX112, QX198, QX172, QX92, QX114, QX97, QX2, QX77, QX132, QX51, QX153, QX192, QX166, QX15, QX20, QX33, QX55, QX98, QX42, QX204, QX88, QX75, QX165, QX63, QX104, QX45, QX84, QX101, QX154, QX117, QX135, QX176, QX47, QX162, QX190, QX16, QX148, QX149, QX121, QX164, QX173, QX13, QX66, QX180, QX188, QX207, QX183, QX11, QX26, QX83, QX113, QX118, QX22, QX181, QX125, QX93, QX95, QX25, QX82, QX44, QX103, QX224, QX202, QX237, QX4, QX189, QX43, QX194, QX102, QX12, QX107, QX90, QX79, QX28, QX196, QX80, QX236, QX14, QX163, QX133, QX178, QX48, QX38, QX10, QX71, QX136, QX5, QX64, QX124, QX120, QX40, QX68, QX138, QX73, QX203, QX70, QX34, QX87, QX122, QX216, QX110, QX8, QX106, QX6

Reagents to generate NILs:

Strain	Genotype	Constructed from	Left primer	Right primer
ECA573	eanIR322[chrV; CB>N2]	N2xCB4856	oECA1141 & oECA1142	oECA1147 & oECA1148
ECA554	eanIR321[chrV; N2>CB]	N2xCB4856	oECA1141 & oECA1142	oECA1147 & oECA1148
ECA1059	eanIR434[chrV; CB > N2]	ECA334xN2	oECA1141 & oECA1142	oECA741 & oECA742
ECA1065	eanIR440[chrV; CB>N2]	N2xCB4856	oECA1141 & oECA1142	oECA1147 & oECA1148
ECA377	eanIR163[chrV, CB>N2]	N2xECA290	oECA799 & oECA800	oECA801 & oECA802
ECA232	eanIR152[chrV; CB>N2]	QX450xN2	oECA799 & oECA800	oECA745 & oECA746
ECA629	eanIR337[chrV; CB>N2]	ECA514xN2	oECA759 & oECA760	oECA775 & oECA776
ECA634	eanIR342[chrV; CB>N2]	ECA518xN2	oECA759 & oECA760	oECA775 & oECA776
ECA632	eanIR340[chrV; CB>N2]	ECA516xN2	oECA759 & oECA760	oECA775 & oECA776
ECA636	eanIR344[chrV; CB>N2]	ECA521xN2	oECA759 & oECA760	oECA775 & oECA776

Primers

**Primer**	**Genomic position**	**Sequence**
oECA1141	V:144,547	ctcatgggagtaacctgggc
oECA1142	V:144,547	cggtgacaacggagaatcca
oECA1147	V:20,622,851	gtttagtaccagcggggcat
oECA1148	V:20,622,851	tgcattccgacccaagagac

oECA741	V:11,940,588	ccagaatttagcatgcgtggg
oECA742	V:11,940,588	agtgtctggttccgttagtact
oECA799	V:7,862,556	ttctcgctactggaacacgc
oECA800	V:7,862,556	tcaagaagcgttgggaagtct
oECA745	V:13,110,045	tgcagaggtggagtaaccct
oECA746	V:13,110,045	ctcggtctctcccccactaa
oECA759	V:5,730,447	tgttccggcaagttctcgaa
oECA760	V:5,730,447	gctgcagccggaaattgttt
oECA775	V:8,373,117	acacccttcttttgcggaca
oECA776	V:8,373,117	gtctgttgcgcatgtttcgt
